# Wild inside: Urban wild boar select natural, not anthropogenic food resources

**DOI:** 10.1371/journal.pone.0175127

**Published:** 2017-04-12

**Authors:** Milena Stillfried, Pierre Gras, Matthias Busch, Konstantin Börner, Stephanie Kramer-Schadt, Sylvia Ortmann

**Affiliations:** 1 Department of Evolutionary Ecology, Leibniz Institute for Zoo and Wildlife Research IZW, Alfred-Kowalke-Strasse 17, Berlin, Germany; 2 Department of Animal Ecology and Tropical Biology, Julius Maximilians Universität Würzburg, Am Hubland, Wuerzburg, Germany; Universidade de Aveiro, PORTUGAL

## Abstract

Most wildlife species are urban avoiders, but some became urban utilizers and dwellers successfully living in cities. Often, they are assumed to be attracted into urban areas by easily accessible and highly energetic anthropogenic food sources. We macroscopically analysed stomachs of 247 wild boar (*Sus scrofa*, hereafter WB) from urban areas of Berlin and from the surrounding rural areas. From the stomach contents we determined as predictors of food quality modulus of fineness (MOF,), percentage of acid insoluble ash (AIA) and macronutrients such as amount of energy and percentage of protein, fat, fibre and starch. We run linear mixed models to test: (1) differences in the proportion of landscape variables, (2) differences of nutrients consumed in urban vs. rural WB and (3) the impact of landscape variables on gathered nutrients. We found only few cases of anthropogenic food in the qualitative macroscopic analysis. We categorized the WB into five stomach content categories but found no significant difference in the frequency of those categories between urban and rural WB. The amount of energy was higher in stomachs of urban WB than in rural WB. The analysis of landscape variables revealed that the energy of urban WB increased with increasing percentage of sealing, while an increased human density resulted in poor food quality for urban and rural WB. Although the percentage of protein decreased in areas with a high percentage of coniferous forests, the food quality increased. High percentage of grassland decreased the percentage of consumed fat and starch and increased the percentage of fibre, while a high percentage of agricultural areas increased the percentage of consumed starch. Anthropogenic food such as garbage might serve as fallback food when access to natural resources is limited. We infer that urban WB forage abundant, natural resources in urban areas. Urban WB might use anthropogenic resources (e.g. garbage) if those are easier to exploit and more abundant than natural resources. This study shows that access to natural resources still is mandatory and drives the amount of protein, starch, fat or fibre in wild boar stomachs in urban as well as rural environments.

## Introduction

Urban areas are expanding worldwide, thereby fragmenting habitats and threatening biodiversity [1]. While urban avoiders [2,3] are “losers” of urbanization, urban utilizers and dwellers [2,3] succeed even in cities where artificial landscape structures [4] lead to a decrease in biodiversity [5]. In addition to alterations in the landscape, urban animals have to deal with human disturbances [6,7] which are often happening at small-scales, with animals being able to distinguish spatial variations in risk [8–10]. To succeed in urban areas, animals have to trade-off between access to food and predator avoidance including anthropogenic disturbance [11,12]. Increasing the tolerance towards disturbances is one way of trading-off fear vs. food and can be determined by ecological, behavioural, and physiological characteristics such as habitat selection, metabolic rate and ingestion rate [13].

Optimal access to food depends on the type of food, optimal patch choice and time management [14–19] and changes according to highly dynamic resource availability in urban areas as urban landscapes provide natural as well as anthropogenic food sources. Green areas in cities might harbour a high biodiversity [5] and provide natural food items together with street trees or forest patches in cities [20,21]. While studies showing the impact of natural food sources on the diet of urban animals are rare, numerous studies describe the impact of anthropogenic food and garbage [22–26]. Anthropogenic food sources are easily accessible [22,25,27,28] and provide a high amount of energy [29,30]. Increased food availability in urban areas can lead to lower seasonal constraints, resulting in population growth and further expansion into cities [29]. Stomach content analyses revealed that human-associated food supply was sufficient to feed a higher number of animals than currently present, which could explain a continual increase of urban population densities [31]. In another study an inter- and intra-annual variation of foraging pattern was observed. The observed animals showed extensive foraging in urban areas when natural food production was poor and switched back to natural food sources when available [26]. To sum up, urban habitats may provide diverse food sources, and especially opportunistic foragers can benefit from it.

The WB is an omnivorous species with a flexible diet being herbivorous, predacious and granivorous simultaneously [32]. As a versatile forager it displays four main feeding behaviours: browsing and grazing, foraging on the ground, rooting and predation [32]. Foraging WB often get into conflicts with humans, causing intense damage to crops [33–37] and green space [38–40]. WB prefer herbal food over animal food [32,36,41] with a special preference of highly digestible and nutritious food such as acorn, the fruits of the downy oak (*Quercus spec*.)[42]. Among agricultural crops the preferred food of WB is maize (*Zea maize*) [36]. In general, seasonal, inter-annual and regional differences in the diet indicate that WB feed different food types according to availability [41]. Since food availability in urban and rural areas differs due to the high availability of anthropogenic food in urban areas [22–26], we expect to find significant differences in the diet of WB from urban areas and those of rural areas.

Small-scale influences can be very important. For example WB in Barcelona are regularly fed in urban areas [22] while in Berlin feeding of wild boar is rare [43]. The Senate of Berlin forbade wildlife feeding, but the effect of this action on WB foraging pattern remains unknown as no monitoring scheme is implemented to date. Therefore, we conduct the first study comparing diets of WB along an urban-rural gradient, which includes urban forests, built up areas and rural areas. We hypothesize that the diets of WB from the respective origins differ and that the diets reflect the characteristic urban and rural food resources of respective patches.

We predicted that

1. urban WB consume a higher amount of anthropogenic food sources than rural WB;
2. different macroscopic stomach types mirror typical landscape composition, since areas of urban WB are dominated by human associated landscape structures while rural WB deal more with natural landscapes;
3. the amount of nutrients and energy are expected to be highest in stomachs of urban WB;
4. landscape structures influence energetic value and quality of food.

## Material and methods

### Study area and sample collection

The study was conducted in Berlin (52°31’N, 13°24’E) and surrounding areas of the Federal State of Brandenburg. Twenty percent of the area of Berlin was covered with forests, divided into four main forests. The forest of the western part of Berlin was reforested with mixed trees (pine *Pinus sylvestris*, oak *Quercus robur* and *Quercus rubra*, and beech *Fagus sylvatica*) after the second world war and afterwards used as recreational forest [44]. The eastern part of the Berlin forest and the forests in Brandenburg are covered with coniferous forests, dominated by pine. Between 2012 and 2015 we collected stomachs of 247 WB and stored the samples frozen at -20°C. The stomachs were collected from single hunts carried out by ‘city hunters’ and from large battue hunts in the urban and rural forests [45].

### Ethical statement

All stomach samples were collected from WB hunted independent of the project, therefore no WB were harmed or killed for the project.

### Macroscopic analysis

For the macroscopic content analysis, the stomach content was spread in a 30x40cm bowl and searched for macroscopic identifiable food residues. All single food residues were recorded qualitatively with the help of a checklist. The stomachs were categorized in five categories according to the dominant food items. A representative subsample of each stomach content was separated for subsequent laboratory analyses.

### Laboratory procedure

To determine the particle size and macronutrients of stomach contents, we first determined dry matter (DM) by drying a subsample of 10 grams in a drying oven (Memmert UM600, Schwabach, Germany) at 100°C for 24h. Another subsample was used for wet sieve analysis with a Retsch VS1000 laboratory sieve analyser (Retsch GmbH, Haan, Germany) with mesh sizes of 16, 8, 4, 2, 1, 0.5, 0.25, 0.125 and 0.063mm (sieves from Retsch GmbH, Haan, Germany). Particles of each fraction were transferred onto pre-weighed Petri dishes, dried at 100°C for 24 h in the drying oven (Memmert UM600, Schwabach, Germany), and weighed after cooling to room temperature in an exsiccator. For the comparison of the proportion of particles passing the finest sieve, the modulus of fineness (MOF) was calculated for each sample [46,47].

The energy [KJ per g of dry matter] of each sample was determined by burning in a bomb calorimeter (C5000 IKA Labortechnik, Staufen, Germany). The acid-insoluble ash (AIA, [%]) was determined from acid treated raw ash which was produced in a muffle furnace (Heraeus Instruments, Bremen, Germany). Nitrogen concentration was measured with a N-analyser (Elementar rapid NIII, Langenselbold, Germany) and the protein content (as percentage) of a sample was calculated by multiplying the nitrogen concentration with 6.25. A fibre analyser (Ankam200, New York, USA) was used for fibre analysis (as percentage). Starch (as percentage) was determined using a laboratory kit (Boehringer, Mannheim, Germany) and a photometer (Tecan sunrise, Crailsheim, Germany). Crude fat (as percentage) was determined with a fat analyser (Gerhardt Soxherm, Königswinter, Germany). Further explanation can be found in Schwarm et al [48].

### Data analyses

#### Analysis of landscape variables

**Fig 1:**
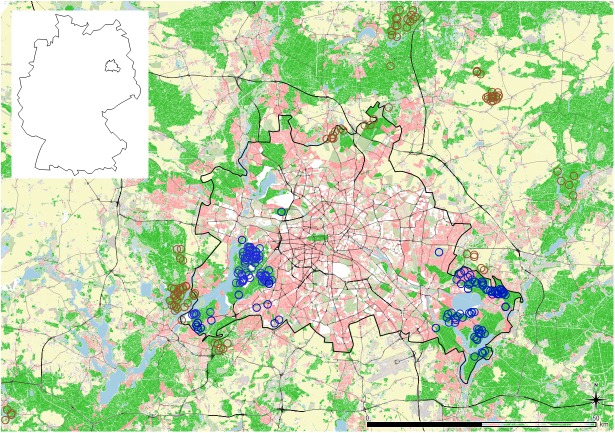
Study area including 2km buffers around sample locations for wild boar stomachs in urban areas of Berlin (n = 151, blue) and rural Brandenburg (n = 96, brown) between 2012 and 2015. All wild boar which were sampled within the geographic border of Berlin were assigned to the urban groups (blue circles). If individual buffers (circles) cross the border between Berlin and Brandenburg, the individuals were assigned to the rural group (brown circles). The black line shows the border of Berlin. The border of Germany and the position of Berlin are shown in the upper left corner of the Fig. Background map: Habitat map of Berlin and Brandenburg, Stillfried et al. unpublished data.

Sample locations for each stomach were imported into QGIS (version 2.14.1, QGIS-Development-Team, Essen, Germany) and a buffer of 2 km^2^ area was calculated around each location (Fig 1). All samples with buffers within the border of Berlin were grouped as urban (n = 151), samples from the surrounding rural areas and those with a buffer that cuts the border between Berlin and Brandenburg were assigned to the rural group (n = 96). The buffer size was based on average home range sizes of GPS-tracked WB within the urban part of the study area [49]. The percentage of different habitat types (grassland, agricultural area, deciduous and coniferous forest and houses) was calculated for each buffer using a reclassified land cover map ([50], for more information see Supplement 1). Additionally, a human population density map [51] was used to calculate mean human density per km^2^ for each buffer. The percentage of sealing was calculated for each buffer by using the extract function in the statistical software R (version 3.3.1 [52]) using a 100x100m raster map, which shows sealed surfaces ([53], for more information see S1 Supplement).

#### Macroscopic analysis

For the macroscopic analysis, we counted and displayed every single anthropogenic food item (due to the small amount of anthropogenic food sources, statistical analyses were omitted). To test the distribution of stomach categories within urban and rural WB, we used a χ^2^-test and plotted the results in a mosaic plot. The colours used urban (blue) and rural (brown) are the same as used in Fig 1.

#### Landscape in rural and urban areas

The percentage of human related landscape variables (sealed soil, houses and human density, see Table 1 for variable description), forest related landscape variables (deciduous and coniferous) and agricultural areas (grassland and agriculture) among urban and rural origins were tested with linear mixed models (LMMs, MuMin package [54]). Models including each of the previously listed variables as response, origin (rural vs. urban) as fixed effect, as well as month and forestry district as crossed random factors, were compared to the corresponding null model. We used information criteria (Akaike’s Information criterion corrected for small sample sizes, AICc and Bayesian Information Criterion, BIC) to check whether the final model was superior to an intercept-only model. Models were considered similar if differences in AICc were less than 2.5 (Hilbe 2009); as the evaluation of our models with all the information criteria produced similar conclusions, we further discussed only AICc values. Significance of level difference—within the predictor variable ‘origin’—was determined by the Tukey post-hoc test using significance level of 0.05 (function glht in library multcomp [55]).

**Table 1:** Overview of variables which were used for linear mixed models, analysing wild boar stomach contents in Berlin and Brandenburg between 2012 and 2015. A first set of models was testing the variation af landscape variables within different spatial areas and in a second model set, nutrient values and how they vary amoung groups of origin, among different stomach content categories and in relation to landscape variables.

Name	Description
**Origin**	***SPATIAL VARIABLE***: origin of wild boar:
	Rural group, Brandenburg
	Urban group, city and forests of Berlin
**Stomach Category** **(SC)**	***MACROSCOPIC VARIABLE*:** regarding the most dominant contents:
	Acorn–including only Acorn and grubs
	Acorn /Fibre–Mix of different fibre types and acorn
	Fibre–only fibre
	Maize–mostly maize, but mixed with several other contents
	Mix–when none of the above groups fitted
**Sealing**	% of sealed surfaces-human associated variable
**Houses**	% of buildings + house with garden -human associated variable
**Human Density**	Human density (HumDens) per km^2^ -human associated variable
**Decidous**	% of decisuous forests within each wild boar area- forest variable
**Coniferous**	% coniferous forests within each wild boar area- forest variable
**Grassland**	% of public and private grasslands–agricultural variable
**Agriculture**	% of agricultural area–agricultural variable
**Month**	Temporal random factor: month when samples was collected.
**Forest area**	Spatial random factor: forest area where the sampel was collected.

#### Macronutrients among rural and urban origin and stomach categories

In a second model set, we used LMMs to test energy, MOF, AIA, protein, starch, fat or fibre as response variable and the origin and stomach category as explanatory variable. Model configuration, selection and determination of significant differences of levels among origin- and stomach-category were conducted as described above.

#### Impacts of landscape variables on macronutrients

In a third set of models (LMM), the abovementioned landscape variables were used as fixed effects, using energy, MOF, AIA, percentage of protein, starch, fat and fibre as response (Table 1). We compared a set of candidate models (S1 Table) for each response variable. While a “Full” model included all landscape variables; the models “Hum1 to Hum4” contained only human related landscape variables; the “For1 to For3” models contained only forest variables and the “Agr1 to Agr3” models contained only grassland and agriculture variables (list of candidate models in S1 Table). The “null” model contained only the random effects. Prior to LMM fitting we tested the explanatory variables for correlation, and only variables under 0.7 were retained (S1 Fig). Due to multicollinearity of landscape variables in the different origin categories for the response variable energy, we split this analysis and ran a separate model set for urban and rural. There was no residual multicollinearity within used models (calculated with the function vif.mer, adapted from rms::vif, downloaded from https://raw.githubusercontent.com/aufrank/R-hacks/master/mer-utils.R). No multicollinearity was present among variables used in each of the models. For determination of the final model, we calculated the relative variable importance using the function model.sel from the MuMIn package [54] using all model with a delta AICc below 2. Variables with a relative variable importance above 0.4 were included in the final model and the effects of those variables were visualized in R using the effects-package [56].

## Results

### Macroscopic analysis

#### Anthropogenic food items and stomach content categories

Sixteen out of 247 of the WB stomachs used for the macroscopic analysis contained potential anthropogenic food (S2 Table). Five stomachs contained apples. Three apple-stomachs were collected in rural areas as well as two in urban areas between December and February. Four wild boar from urban areas also consumed bread, two sausages or cheese and five pieces of plastic.

All other stomachs contained only natural food and were assigned to one of the following five categories: (1) The “acorn-fibre-stomach” consisted of mostly acorn and different types of fibre with different quantitative compositions; (2) the “acorn-stomach” contained mostly acorn, often mixed with cockchafer grubs, but no fibre; (3) the “fibre-stomach” contained mostly fibres, roots and reed; (4) the “maize-stomach” contained a high amount of maize, often mixed with acorn but no fibres. (5) All the stomachs that did not fit into one of the described category where labelled as “mix-stomach”.

#### Stomach categories vs. origin

We found no significant difference for the distribution of stomach categories among rural and urban origin (S2 Fig, Pearson’s Chi squared test, X^2^ = 6.21, df = 4, p = 0.18, Phi = 0.16, n = 247). All five stomach categories were present in both, urban and rural areas.

### Landscape within urban and rural origin

**Fig 2:**
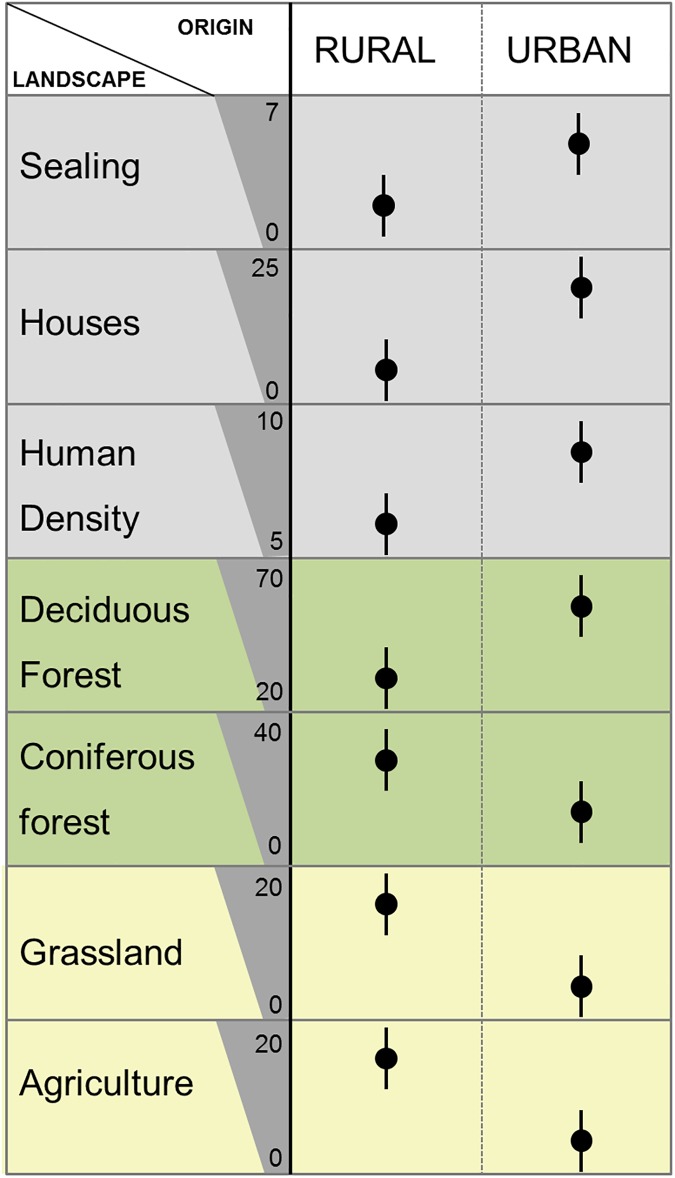
Percentage of different landscape variables among urban wild boar from Berlin and rural wild boar from Brandenburg between 2012 and 2015. Landscape variables are either human associated landscape variables (grey shade) such as percentage of sealed area, percentage of houses or human density within a buffer of 2 km^2^; forest associated landscape variables (green shade) include percentage of deciduous forest and percentage of coniferous forest; agriculture associated variables (yellow) are percentage of grassland and agricultural area. Significant difference (rural vs. urban) was determined by Tukey post hoc test and indicated with different characters. (a-b, S4 Table). Vertical lines show the 95% confidence intervals.

The model selection revealed that all the tested landscape variables differed among urban and rural landscapes, as the model including origin as fixed effect was the best model in all model sets (S3 Table). The conduced post-hoc test proved significant differences among levels for all landscape variables except human density (S4 Table): In detail, percentage of sealing was 6% in the urban and 2% in the rural group. Percentage of houses was highest in the urban group (24%) compared to 8% in the rural group (Fig 2). Percentage of deciduous forest was significantly higher in the urban group (60%) than in the rural group (20%), while the coniferous forest was more dominant in rural areas (35% versus 10% in the urban group). Grassland and agriculture area were also higher in rural landscapes (18% grassland and agricultural area) than in the urban group (5% grassland, 4% agriculture, Fig 2).

### Macronutrients among rural and urban origin

**Fig 3:**
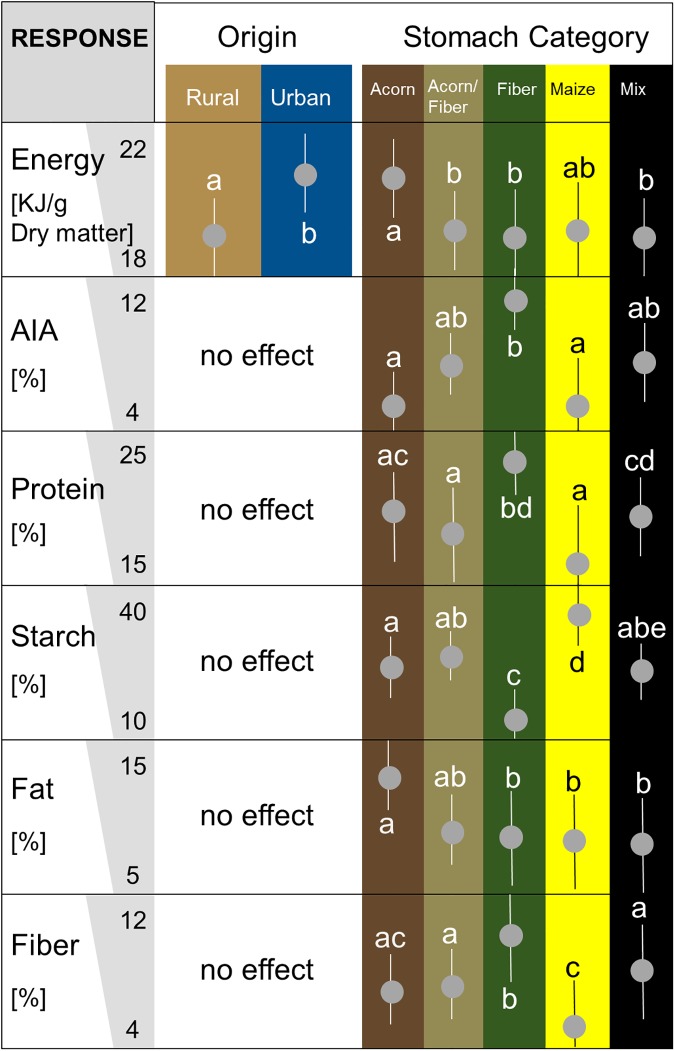
Variation of macronutrients of wild boar from Berlin and Brandenburg between 2012 and 2015 among groups of different origin and among stomach content categories. The energy amount of each stomach content was measured in KJ/g dry matter, the acid insoluble ash (AIA) is given in percent dry matter, such as amount of protein, starch, fat and fibre. Significant differences of origin were indicated using brown (rural) or blue (urban) background. For similar pattern we wrote “no effect”. Differences between wild boar stomach categories “Acorn (dark brown), Acorn/Fibre (olive green), Fibre (green), Maize (yellow), Mix (black)”were tested by Turkey post hoc test (S6 Table). Significant differences of levels of each category were visualized by labeling with characters a-e; different characters indicate significant differences. Vertical lines show 95% confidence intervals. Model selection table: S5 Table.

For all candidate model sets, except the MOF, the best model was the model including ‘Origin’ (S5 Table). The post-hoc test revealed significant differences among rural and urban landscapes for Energy (S6 Table). The Energy amount was significantly higher in urban landscapes (21KJ/g, Fig 3) than in rural areas (18KJ/g, Fig 3). The food items from acorn stomachs (21.5KJ/g) contained significantly more energy than fibre and acorn-fibre stomachs but similar amounts to maize stomachs. AIA values were lowest in acorn (4%) and maize (4%) stomachs and highest in fibre stomachs (12%, Fig 3, S6 Table). Protein was highest within the fibre stomachs (25%) whereas the maize stomach showed the lowest protein value (15%). The percentage of starch within maize stomachs was highest (40%) and lowest in fibre stomachs (10%). Acorn stomachs contained 14% fat, while other stomach categories had about 10% fat. The percentage of fibre was also highest within fibre stomachs (11% Fig 3, S6 Table).

### Impacts of landscape variables on macronutrients

**Fig 4:**
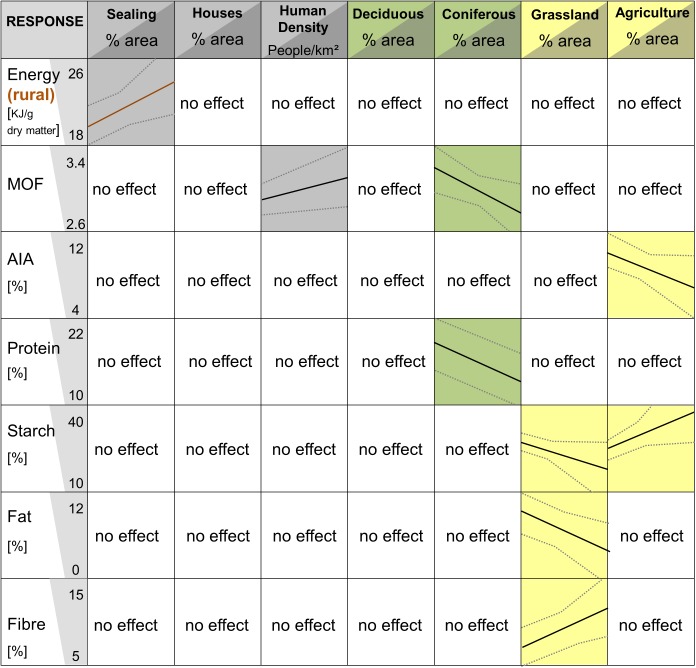
Variation of macronutrients of wild boar from Berlin and Brandenburg between 2012 and 2015 in relation to different landscape structures. Here we present only variables with a relative variable importance above 0.4 (S7 Table). The response energy was analyzed separately for rural and urban wild boar; the only effects shown results from rural wild boar (brown line). For the other variables all samples were used. Colours indicate the variable groups: Human associated (grey), forest associated (green), agriculture associated (yellow). For each panel of the compound Fig the x-axis show the values for the nutrients and the y-axis the percentage cover of each land-use category within a buffer (increasing from left to right) the continuous lines show magnitude of change (slope) due to changing share of the landscape, the dashed lines indicate the 95% confidence intervals. (See associated model selection table: Table 2, S8 Table).

Model selection resulted in several equally well fitted models having an AICc below 2.5 (Table 2). The relative variable importance (S7 Table) showed the variables with the highest impact, which were sealing for energy in rural areas; human density and coniferous forest for MOF; agriculture for AIA; coniferous forest for protein, agriculture and green area for starch; and green area for fat and fibre (S7 Table). The amount of energy increased (from 18 to 23%) with increasing percentage of sealing in rural areas (Fig 4). No effect was found for landscape variables and energy in urban area. The MOF increased (from 2.8 to 3.2%) with human density and decreased (from 3.3 to 2.7) with increasing percentage of coniferous forests fell (from 20% to 13%) with increasing percentage of coniferous forests. In areas with high proportions of agriculture, the percentage of starch became higher (from 20% to 40%) but decreased (from 30% to 15%) with increasing percentage of grassland, Fat decreased from 10% to 4% and fibre increased from 7% to 12% with increasing percentage of grassland.

**Table 2:** Model selection table for linear mixed models, testing nutrient values and food quality in stomachs of wild boar from Berlin and Brandenburg between 2012 and 2015.

Response		Model	Intercept	Sealing	Houses	Human density	Deciduous	Coniferous	Grassland	Agriculture	df	logLik	AICc	delta	BIC
**Energy**	Hum2		19.14	0.67							5	-234.87	480.41	0.00	492.56
**rural**	Hum1		19.21	0.75	-0.41	-0.38					7	-233.01	481.29	0.88	497.96
**Energy**	null		19.40								4	-341.78	691.83	0.00	703.62
**urban**	For2		19.27				-0.21				5	-341.21	692.83	1.00	707.50
	For3		19.38			0.18					5	-341.35	693.11	1.28	707.78
	Agr2		19.47					-0.15			5	-341.48	693.38	1.55	708.05
	Agr3		19.47		-0.13						5	-341.58	693.57	1.74	708.24
	Hum4		19.39	0.10							5	-341.63	693.68	1.85	708.34
**MOF**	For3		2.98					-0.08			5	-191.46	393.16	0.00	410.32
	Hum4		2.96			0.06					5	-191.89	394.02	0.86	411.32
	Hum1		2.96	-0.04	0.05	0.07					7	-190.14	394.76	1.59	418.85
	Agr2		2.96						0.05		5	-192.35	394.95	1.79	412.24
**AIA**	Agr3		8.48							-1.10	5	-853.16	1716.57	0.00	1733.86
	Agr1		8.42						0.53	-1.11	6	-852.61	1717.58	1.01	1738.28
	null		8.18								4	-854.86	1717.89	1.32	1731.75
**Protein**	For3		18.01					-1.35			5	-749.85	1509.95	0.00	1527.24
	Full		18.06	-0.31	-1.00	0.14	-1.54	-2.27	-0.78	-1.12	11	-744.35	1511.82	1.87	1549.30
**Starch**	Agr1		24.10						-1.80	2.14	6	-995.49	2003.33	0.00	2024.03
	Full		24.10	0.64	1.96	-1.01	3.89	3.57	0.05	2.90	11	-991.01	2005.15	1.82	2042.63
**Fat**	Agr2		9.00						-1.00		5	-850.98	1712.22	0.00	1729.51
	Hum4		8.82			0.75					5	-851.83	1713.90	1.69	1731.20
	Agr1		8.89						-1.00	0.23	6	-850.89	1714.13	1.92	1734.84
**Fibre**	Agr2		9.30						0.57		5	-750.08	1510.41	0.00	1527.71
	null		9.31								4	-751.61	1511.39	0.98	1525.26
	For3		9.55					-0.55			5	-750.93	1512.11	1.69	1529.40
	Agr1		9.33						0.57	-0.15	6	-749.99	1512.33	1.92	1533.03

For different response variables, the energy amount of each stomach content was measured in KJ/g dry matter. Only the analysis of energy was split into urban and rural origin because Fig 3 showed a significant difference between urban and rural wild boar only for energy. The modulus of fineness (MOF) was calculated after particle size determination; the acid insoluble ash (AIA) is given in percent, such as amount of protein, starch, fat and fibre.

The explanatory variables describe the landscape within a buffer around each sample location and were grouped regarding their expected influence: Sealing (percentage of sealed surface), houses (percentage of houses) and HumDens (Human density per km^2^) are human associated landscape variables. The Models, which include only these variables, are called “Hum1”-“Hum4”. Deciduous (percentage of deciduous forest) and Coniferous (percentage of coniferous forest) are forest associated landscape variables; the models which include only these variables are called “For1”-“For3”. Grassland (percentage of grassland) and Agriculture (percentage of agriculture) are agricultural associated landscape variables; the model which include only these variables are called “Agr1”-“Agr3”. The full model includes all variables; the intercept only model is called “null”.

The degree of freedom is abbreviated as “df”. The logarithmic likelihood is abbreviated as “logLik”. Akaike’s information criterion corrected for small sample size (AICc) is used for model selection, such as the Bayesian information criterion (BIC). The delta shows the difference between the AICc values. Only models with a delta AICc below 2 are displayed here. Full model selection table in S8 Table.

## Discussion

### Anthropogenic food sources

The general assumption that WB enter cities to primarily consume human garbage or receive direct feeding (prediction 1) needs to be reconsidered, at least in regions restrictive supplementary wildlife feeding rules. Nevertheless, urban WB consume food of higher quality than their rural counterparts, because local landscapes provide different resources to satisfy nutritional needs.

Contrasting to our findings, WB from less restrictive managed regions (Barcelona or Islamabad) frequently consumed anthropogenic food [22,23]. Even if our study underestimated amounts of anthropogenic food, as some food items get digested more rapidly [41,57] or some plant fibres might origin from human composts and were not identifiable as human-associated, the amount of natural food still predominates anthropogenic food.

Comparable omnivores such as black bears foraged intensively in urban areas when natural food production was poor, but switched to natural food sources whenever available [26]. However, black bears in Montana foraged on human foods near houses even when natural foods were available [58]. Coyotes in Chicago consumed human-associated food during pup-rearing and dispersal seasons, i.e. when energy demands are high [59]. Most of our WB stomach samples were collected during late autumn/winter, when a high amount of mast was available. Mast is the preferred and most dominant food in wild boar when available [41,60,61]. Since WB switch diets seasonally and depend on availability [41,60,62] our results show a typical winter pattern. Due to a lower availability of mast and higher human activity in urban forests (higher availability of garbage in form of leftovers from recreational activities), we would expect a higher amount of anthropogenic food sources in summer. In addition, rooting damage of WB in urban areas of Berlin were higher in summer (personal observation) which might be a result of higher presence in urban areas in summer. To fulfil their energy requirements WB might then also increase the consumption of garbage, if available. It might be possible that the local WB preferred natural food sources by choice. Another possibility might be that anthropogenic food sources were difficult to access in our study region, as the Berlin senate campaigned to inform people that supplementary feeding wildlife is illegal (detailed information and Flyer on the homepage of Berlin: http://www.stadtentwicklung.berlin.de/forsten/wildtiere/download/fuettern_nein_danke.pdf). and the Berlin forestry department removed all garbage bins from the forests to reduce the amount of garbage left by people [63,64]. In Colorado, bear-resistant garbage containers existed, but more than 50% were not properly secured [26]. Baboons (*Papio ursinus*) in South Africa showed a strong preference for anthropogenic food, thus fencing of waste sites resulted in a decreased appearance of baboons in urban areas [65]. WB can function as potential reservoir for pathogenic zoonoses such as hepatitis E or leptospirosis which were detected in Berlin [66,67] and WB from Barcelona even carried antimicrobial resistances after feeding on human garbage [68]. As long as WB from Berlin consume natural food sources, it is unlikely that they get into contact with antimicrobial resistances from human origin and that they function as carrier. But an increased potential for zoonotic diseases might be a consequence if the highly flexible omnivores switch their diets towards anthropogenic food in relation to food availability [41,60,62]. Therefore we infer limited accessibility of human garbage as a viable management tool. Additionally, combined with public education, it can be even more effective in preventing wildlife conflicts, especially in respect of potential disease transmissions.

### Stomach categories and typical landscape composition in urban and rural areas

The five stomach categories found in WB of our study area were comparable with major herbal food categories reported for WB [41] consuming mostly plant material [32,60,69–71]. However, the stomach types do not mirror a typical landscape composition (prediction 2, part1), because our results show a clear differentiation in the landscape composition of urban and rural areas (prediction2, part 2) which is not reflected in the frequency of stomach types in urban and rural areas. This shows that the omnivorous WB [36] feeds various food items, but is selective for high forage quality and high carbohydrate contents [70]. WB diet is a result of environmental characteristics and resources [36]. But due to specific behavioural patterns, we infer that food quality and available energy is also a matter of food choice if different resources are available. Our results therefore prove that urban areas—even though dominated by anthropogenic structures–can provide enough natural resources to enable natural feeding patterns and even offering higher amounts of energy than natural food in rural areas.

### Nutrients and energy in relation to origin and food type

An optimal access to food, as described by optimal foraging theory, depends on the food choice, optimal patch choice and time management [14–19]. Our prediction (3) that energy is highest in stomachs of urban wild boar could be confirmed by our results. The observation that the composition of nutrients is independent from the origin and only varies among the consumed food items (represented by stomach categories) is in line with the above-mentioned observation that WB select for high quality food in whatever environment. Acorns contain a high amount of fat [72]. The observed relatively high percentage of protein in acorn-stomachs, despite acorn containing low amounts of protein [72], fits very well, as acorn was often consumed together with a large number of chockhafer grubs providing necessary protein [38]. Numerous studies describe that WB forage within agricultural areas and are crop pests [33–37,41,71,73]. Maize is often consumed [36] and is known for its high amount of carbohydrates. Interestingly, we found high amount of maize-stomachs in urban areas, but relatively few agricultural areas. We assume, most of the maize consumed in urban areas is a result of anthropogenic supplemental food [74,75], as maize as hunting bait is frequently used and not prohibited in the study area.

A high MOF value indicates high fibre content and therefore poor nutritional quality because fibre provides little energy and is difficult to digest [76,77] which was the case in our study in areas with high human density with is contradictory to the observation that energy increased with high percentage of sealing. Acid insoluble ash (AIA) indicates the amount of indigestible soil which is unintentionally ingested by feeding wildlife [78]. Rooting WB might ingest more soil; hence, we considered the amount of AIA as an indicator of rooting intensity within a landscape. Therefore our results show that foraging in agricultural areas occurs mostly above ground.

### Impact of landscape on food quality

The landscape structure influences energy and quality of available food for WB. While the effect of urbanization (increase of human associated landscape variables) has a larger impact for rural WB, regarding the amount of energy, human associated landscape variables play a minor role for the quality of food, as represented by the MOF. A higher food quality in urban areas, as decribed by other studies [29,30] is therefore independent from the landscape per se, but depends on the interaction with humans [22–26]. Deciduous forests had no and coniferous forests only a weak impact on the nutrient composition. These results suggest that areal shares of resources within a home range (here 2km^2^) are little important, as long as WB have access. Since urban areas harbour a high percentage of deciduous forests providing highly energetic acorns, WB can use adjacent patches as described by the optimal patch choice theory [14,15,19]. Finally, we infer mobile WB are great urban invaders and urbanites, as their trophic plasticity enables them to satisfy nutritional needs using various resources. Moreover, wild boar can adjust their behaviour to access and select most suitable resources such as undisturbed and little competed natural resources (e.g. acorn trees) in urban environments.

## Conclusion

In general, WB in urban areas use anthropogenic food such as garbage as fallback food when access to natural resources is limited. Even though urban landscapes strongly dominated by anthropogenic landscape variables, macroscopic stomach types do not mirror the overall landscape. The stomach types indicate a selective food choice within different natural landscape structures. The quality of the consumed food therefore does not depend on the origin per se, but on local characteristics and presence of natural patches to forage. Our results showed wild boar forage abundant, natural resources within urban areas, hence we assume it became more tolerant to disturbance by human urbanites. Moreover, free ranging wild boar switch diets only using anthropogenic resources (e.g. garbage) easy to exploit and more abundant than natural resources.

WB can benefit of anthropogenic landscape structures, where they can find highly energetic resources. Whatsoever, access to natural resources is mandatory and drives the amount of protein, starch, fat or fibre in wild boar stomachs in urban as well as rural environments.

## Supporting information

S1 Supplement Description of geographic database. (PDF)

S1 Fig Correlation plot to test correlation between landscape variables (Pearson’s). Percentage of agriculture (Agr), deciduous forest (DF), coniferous forest (CF), grassland (GL), houses (Ho), Sealing (Se) and human density (HD) were tested. If values are below 0.7, there is no correlation and variables can be used in the same model.
(PDF)

S2 Fig Distribution of wild boar stomach categories from urban areas of Berlin (n = 151, blue box) and rural Brandenburg (n = 96, brown box) between 2012 and 2015. Wild boar stomachs were assigned to the stomach categories Acorn (dark brown), Acorn/Fibre (olive green), Fibre (green), Maize (yellow), Mix (black)“, due to most dominant content, related to a macroscopic stomach content analysis. The size of the stomach category boxes changes in relation to the number of stomachs which belong to a category: The horizontal width represents the sample size (comparison of rural and urban), the vertical width shows the percentage of each category within an origin. In addition, the numbers of stomachs which belong to each category are written within the plot in grey. Results of Pearson’s Chi-squared test: X^2^ = 6,21, df = 4, p = 0.18, Phi = 0.16, n = 247.
(PDF)

S1 Table List of candidate models for linear mixed models, testing the impact of landscape variables on the nutrient composition of wild boar. Seven sets of models were run using the following response variables: energy amount of each stomach content (measured in KJ/g dry matter); modulus of fineness (MOF, calculated after particle size determination); the acid insoluble ash (AIA given in percent), such as amount of protein, starch, fat and fibre. The explanatory variables describe the landscape within a buffer around each sample location and were grouped regarding their expected influence: Sealing (percentage of sealed surface), houses (percentage of houses) and HumDens (Human density per km^2^) are human associated landscape variables (grey). The Models, which include only these variables, are called “Hum1”-“Hum4”. Deciduous (percentage of deciduous forest) and Coniferous (percentage of coniferous forest) are forest associated landscape variables (green); the models which include only these variables are called “For1”-“For3”. Grassland (percentage of grassland) and Agriculture (percentage of agriculture) are agricultural associated landscape variables (shaded in yellow); the model which include only these variables are called “Agr1”-“Agr3”. The full model includes all variables; the intercept only model is called “null”.
(PDF)

S2 Table Anthropogenic food in urban wild boar. Macroscopic stomach content analysis for 247 wild boar in urban areas of Berlin and in rural Brandenburg were conducted between 2012 and 2015. Stomach contents of potential anthropogenic origin are listed here in total and separated into urban origin (blue, n = 151) and rural origin (brown, n = 96).
(PDF)

S3 Table Model selection table for testing landscape within groups of different origin (rural and urban). Seven sets of models were run which compared the intercept only model (“Response_null”) and a model which include the Origin as explanatory variable (model called as response). The response variables describe the landscape within a buffer around each sample location. Human associated landscape variables (grey) are Sealing (percentage of sealed surface), houses (percentage of houses) and HumDens (Human density per km^2^); Forest associated landscape variables (green) are Deciduous and Coniferous (percentage of each forest type); Agricultural associated landscape variables (yellow) are Grassland and Agriculture (percentage of each type).
The degree of freedom is abbreviated as “df”. The logarithmic likelihood is abbreviated as “logLik”. Akaike’s information criterion corrected for small sample size (AICc) is used for model selection, such as the Bayesian information criterion (BIC). The delta shows the difference between the AICc values.
(PDF)

S4 Table Tukey posthoc test for models testing landscape within groups of different origin (rural and urban, S2 Table, Fig 3): The response variables describe the landscape within a buffer around each sample location. Human associated landscape variables (grey) are Sealing (percentage of sealed surface), houses (percentage of houses) and HumDens (Human density per km^2^); Forest associated landscape variables (green) are Deciduous and Coniferous (percentage of each forest type); Agricultural associated landscape variables (yellow) are Grassland and Agriculture (percentage of each type). Significance (bolt numbers) between urban and rural categories is given, when lower and upper 95% confidence interval (CI) have the same sign (both + or both -).
(PDF)

S5 Table Model selection table for linear mixed models, testing the impact of origin and stomach category on the nutrient composition of wild boar stomachs. Seven sets of models were run which compared the intercept only model (“Response_null”) and a model which include the Origin as explanatory variable (model called as response). The response variables are energy amount of each stomach content (measured in KJ/g dry matter); modulus of fineness (MOF, calculated after particle size determination); the acid insoluble ash (AIA given in percent), such as amount of protein, starch, fat and fibre. The degree of freedom is abbreviated as “df”, the logarithmic likelihood is abbreviated as “logLik”. Akaike’s information criterion corrected for small sample size (AICc) is used for model selection, such as the Bayesian information criterion (BIC). The delta shows the difference between the AICc values.
(PDF)

S6 Table Tukey posthoc test for models testing of landscape within groups of different origin (rural and urban, S4 Table, Fig 4): The response variables are energy amount of each stomach content (measured in KJ/g dry matter); modulus of fineness (MOF, calculated after particle size determination); the acid insoluble ash (AIA given in percent), such as amount of protein, starch, fat and fibre. Significance (bolt numbers) between urban and rural categories is given, when lower and upper 95% confidence interval (CI) have the same sign (both + or both -).
(PDF)

S7 Table Relative variable importance for linear mixed models, testing nutrient values and food quality in stomachs of wild boar from Berlin and Brandenburg between 2012 and 2015. Only models with an AICc value below 2 were used to calculate the variable importance. Only variables above 0.4 are used for the final model and visualized in Fig 4.
(PDF)

S8 Table Full model selection table for linear mixed models, testing nutrient values and food quality in stomachs of wild boar from Berlin and Brandenburg between 2012 and 2015. For different response variables, the energy amount of each stomach content was measured in KJ/g dry matter. Only the analysis of energy was split into urban and rural origin because Fig 3 showed a significant difference between urban and rural wild boar only for energy. The modulus of fineness (MOF) was calculated after particle size determination; the acid insoluble ash (AIA) is given in percent, such as amount of protein, starch, fat and fibre.
The explanatory variables describe the landscape within a buffer around each sample location and were grouped regarding their expected influence: Sealing (percentage of sealed surface), houses (percentage of houses) and HumDens (Human density per km^2^) are human associated landscape variables (grey). The Models, which include only these variables, are called “Hum1”-“Hum4”. Deciduous (percentage of deciduous forest) and Coniferous (percentage of coniferous forest) are forest associated landscape variables (green); the models which include only these variables are called “For1”-“For3”. Grassland (percentage of grassland) and Agriculture (percentage of agriculture) are agricultural associated landscape variables (shaded in yellow); the model which include only these variables are called “Agr1”-“Agr3”. The full model includes all variables; the intercept only model is called “null”. The degree of freedom is abbreviated as “df”. The logarithmic likelihood is abbreviated as “logLik”. Akaike’s information criterion corrected for small sample size (AICc) is used for model selection, such as the Bayesian information criterion (BIC). The delta shows the difference between the AICc values.
(PDF)

S9 Table Data table for macroscopic stomach content analysis. A general description of each stomach content is included, as well as presence (1) and absence (0) of specific items.
(TXT)

S10 Table Data table for macronutrient analysis which was used for models. (TXT)
