# Supplementary material for: Wild inside: Urban wild boar select natural, not anthropogenic food resources
Source: PLoS One. 2017 Apr 12;12(4):e0175127. doi: 10.1371/journal.pone.0175127 (PMC5389637; doi:10.1371/journal.pone.0175127)
Supplement: S1 Supplement — (PDF) [file pone.0175127.s001.pdf]

Supplement 1: Description of geographic database. A geographic database for Berlin and Brandenburg was established in order to calculate the percentage of different habitat types within buffers around sample locations of urban mammals in Berlin and Brandenburg. The database contained up-to-date biological habitat maps for Berlin from 2010, downloaded with permission from the "Senatsverwaltung für Stadt und Umwelt" (fisbroker.de), and Brandenburg, downloaded with permission from the website of the Ministry of Rural Development, Environment and Agriculture of the Federal State of Brandenburg (MRDEA, biotop and landuse mapping in Brandenburg, CIR-biotop types 2009), and were merged using ESRI ArcGIS 10.0 (ESRI Inc., Redlands, CA, USA). Since both source maps contained 1,351 habitat categories which were not identical, we reclassified land cover into main habitat categories which the literature suggested would be of interest to understand WB space use. We did this as follows: We separated the classes into the following variables: deciduous forest and coniferous forest, agriculture, water shorelines, swamp, fallow land, industrial, houses with gardens, private and public buildings, private and public green spaces, roads and railways. More details in: Wenzler M (2016) Spatial Classification of Berlins land cover for urban biodiversity assessment- the case of the wild boar. Bachelor thesis, Humbold-University of Berlin, Institute of Geography. For the analysis of wild boar nutrition we only included the following variables in our analyses: Percentage of houses (houses with gardens + private and public buildings), agriculture, grassland (private + public green spaces), deciduous forest, coniferous forest. Additionally, a human population density map downloaded from "Amt für Statistik Berlin-Brandenburg", https://creativecommonsorg/licenses/by/30/de/ was used to calculate mean human density per km² for each buffer. The percentage of sealing was calculated using a "High Resolution Layer: Imperviousness Degree (IMD)"100x100m raster map, which shows sealed surfaces, downloaded from Copernicus Land Monitoring Services - European Environment Agency (EEA), with funding by the European Union.
