# Supplementary material for: Wild inside: Urban wild boar select natural, not anthropogenic food resources
Source: PLoS One. 2017 Apr 12;12(4):e0175127. doi: 10.1371/journal.pone.0175127 (PMC5389637; doi:10.1371/journal.pone.0175127)

S1 Fig: Correlation plot to test correlation between landscape variables (Pearson's). Percentage of agriculture (Agr), deciduous forest (DF), coniferous forest (CF), grassland (GL), houses (Ho), Sealing (Se) and human density (HD) were tested. If values are below 0.7, there is no correlation and variables can be used in the same model.

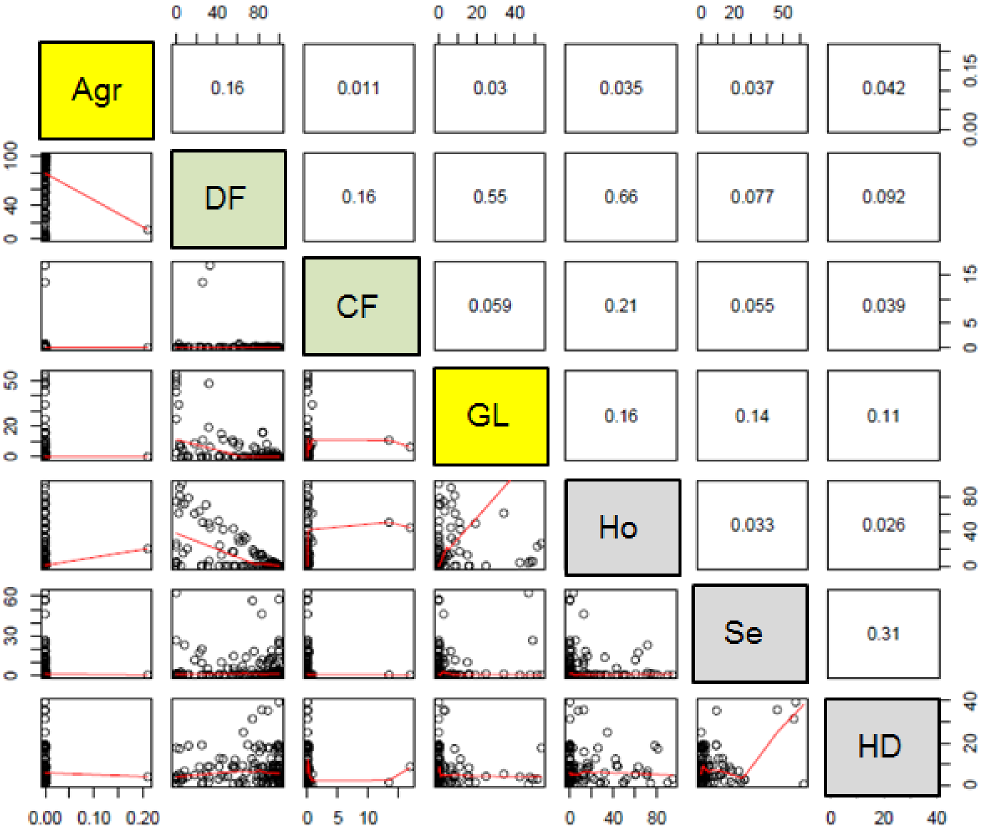

Supplement: S1 Fig — Percentage of agriculture (Agr), deciduous forest (DF), coniferous forest (CF), grassland (GL), houses (Ho), Sealing (Se) and human density (HD) were tested. If values are below 0.7, there is no correlation and variables can be used in the same model. (PDF) [file pone.0175127.s002.pdf]
