# Supplementary material for: Wild inside: Urban wild boar select natural, not anthropogenic food resources
Source: PLoS One. 2017 Apr 12;12(4):e0175127. doi: 10.1371/journal.pone.0175127 (PMC5389637; doi:10.1371/journal.pone.0175127)
Supplement: S2 Fig — Wild boar stomachs were assigned to the stomach categories Acorn (dark brown), Acorn/Fibre (olive green), Fibre (green), Maize (yellow), Mix (black)“, due to most dominant content, related to a macroscopic stomach content analysis. The size of the stomach category boxes changes in relation to the number of stomachs which belong to a category: The horizontal width represents the sample size (comparison of rural and urban), the vertical width shows the percentage of each category within an origin. In addition, the numbers of stomachs which belong to each category are written within the plot in grey. Results of Pearson’s Chi-squared test: X2 = 6,21, df = 4, p = 0.18, Phi = 0.16, n = 247. (PDF) [file pone.0175127.s003.pdf]

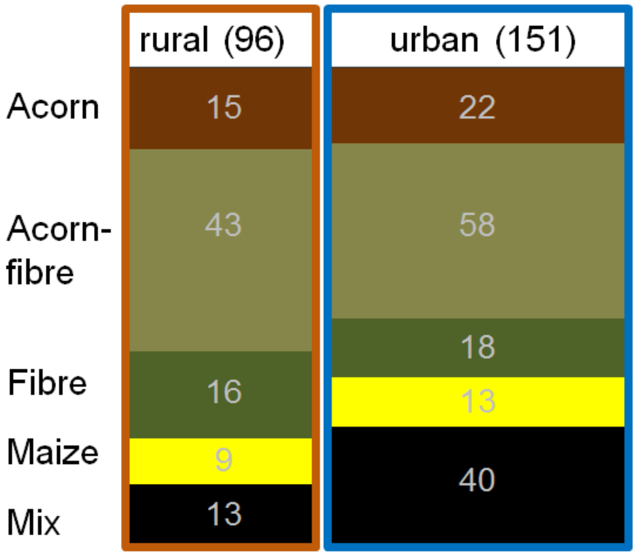

S2 Fig: Distribution of wild boar stomach categories from urban areas of Berlin (n=151, blue box) and rural Brandenburg (n=96, brown box) between 2012 and 2015. Wild boar stomachs were assigned to the stomach categories Acorn (dark brown), Acorn/Fibre (olive green), Fibre (green), Maize (yellow), Mix (black)", due to most dominant content, related to a macroscopic stomach content analysis. The size of the stomach category boxes changes in relation to the number of stomachs which belong to a category: The horizontal width represents the sample size (comparison of rural and urban), the vertical width shows the percentage of each category within an origin. In addition, the numbers of stomachs which belong to each category are written within the plot in grey. Results of Pearson's Chi-squared test: X^2=6,21, df=4, p=0.18, Phi=0.16, n=247.
