# Supplementary material for: Wild inside: Urban wild boar select natural, not anthropogenic food resources
Source: PLoS One. 2017 Apr 12;12(4):e0175127. doi: 10.1371/journal.pone.0175127 (PMC5389637; doi:10.1371/journal.pone.0175127)
Supplement: S1 Table — Seven sets of models were run using the following response variables: energy amount of each stomach content (measured in KJ/g dry matter); modulus of fineness (MOF, calculated after particle size determination); the acid insoluble ash (AIA given in percent), such as amount of protein, starch, fat and fibre. The explanatory variables describe the landscape within a buffer around each sample location and were grouped regarding their expected influence: Sealing (percentage of sealed surface), houses (percentage of houses) and HumDens (Human density per km2) are human associated landscape variables (grey). The Models, which include only these variables, are called “Hum1”-“Hum4”. Deciduous (percentage of deciduous forest) and Coniferous (percentage of coniferous forest) are forest associated landscape variables (green); the models which include only these variables are called “For1”-“For3”. Grassland (percentage of grassland) and Agriculture (percentage of agriculture) are agricultural associated landscape variables (shaded in yellow); the model which include only these variables are called “Agr1”-“Agr3”. The full model includes all variables; the intercept only model is called “null”. (PDF) [file pone.0175127.s004.pdf]

S1 Table: List of candidate models for linear mixed models, testing the impact of landscape variables on the nutrient composition of wild boar. Seven sets of models were run using the following response variables: energy amount of each stomach content (measured in KJ/g dry matter); modulus of fineness (MOF, calculated after particle size determination); the acid insoluble ash (AIA given in percent), such as amount of protein, starch, fat and fibre. The explanatory variables describe the landscape within a buffer around each sample location and were grouped regarding their expected influence: Sealing (percentage of sealed surface), houses (percentage of houses) and HumDens (Human density per km²) are human associated landscape variables (grey). The Models, which include only these variables, are called "Hum1"-"Hum4". Deciduous (percentage of deciduous forest) and Coniferous (percentage of coniferous forest) are forest associated landscape variables (green); the models which include only these variables are called "For1"-"For3". Grassland (percentage of grassland) and Agriculture (percentage of agriculture) are agricultural associated landscape variables (shaded in yellow); the model which include only these variables are called "Agr1"-"Agr3". The full model includes all variables; the intercept only model is called "null".

Model	Response	Human variables	Forest variables	Agricultural variables	Random
Full	Response	Sealing + Houses + HumDens	+ Deciduous + Coniferous	+ Grassland + Agriculture	Month + FA
Hum1	Response	Sealing + Houses + HumDens			Month + FA
Hum2	Response	Sealing			Month + FA
Hum3	Response	Houses			Month + FA
Hum1	Response	HumDens			Month + FA
For1	Response		Deciduous + Coniferous		Month + FA
For2	Response		Deciduous		Month + FA
For3	Response		Coniferous		Month + FA
Agr1	Response			Grassland + Agriculture	Month + FA
Agr2	Response			Grassland	Month + FA
Agr3	Response			Agriculture	Month + FA
null	Response				Month + FA
