# Supplementary material for: Wild inside: Urban wild boar select natural, not anthropogenic food resources
Source: PLoS One. 2017 Apr 12;12(4):e0175127. doi: 10.1371/journal.pone.0175127 (PMC5389637; doi:10.1371/journal.pone.0175127)
Supplement: S2 Table — Macroscopic stomach content analysis for 247 wild boar in urban areas of Berlin and in rural Brandenburg were conducted between 2012 and 2015. Stomach contents of potential anthropogenic origin are listed here in total and separated into urban origin (blue, n = 151) and rural origin (brown, n = 96). (PDF) [file pone.0175127.s005.pdf]

S2 Table: Anthropogenic food in urban wild boar. Macroscopic stomach content analysis for 247 wild boar in urban areas of Berlin and in rural Brandenburg were conducted between 2012 and 2015. Stomach contents of potential anthropogenic origin are listed here in total and separated into urban origin (blue, n=151) and rural origin (brown, n=96).

**Content**	**Total count**	**Total %**	**RURAL (n=96)**	**URBAN (n=151)**
Apple	5	2.02	3	2
Bread	4	1.62		4
Sausage/Cheese	2	0.81		2
Plastic	5	2.02		5
