# Supplementary material for: Wild inside: Urban wild boar select natural, not anthropogenic food resources
Source: PLoS One. 2017 Apr 12;12(4):e0175127. doi: 10.1371/journal.pone.0175127 (PMC5389637; doi:10.1371/journal.pone.0175127)
Supplement: S3 Table — Seven sets of models were run which compared the intercept only model (“Response_null”) and a model which include the Origin as explanatory variable (model called as response). The response variables describe the landscape within a buffer around each sample location. Human associated landscape variables (grey) are Sealing (percentage of sealed surface), houses (percentage of houses) and HumDens (Human density per km2); Forest associated landscape variables (green) are Deciduous and Coniferous (percentage of each forest type); Agricultural associated landscape variables (yellow) are Grassland and Agriculture (percentage of each type). The degree of freedom is abbreviated as “df”. The logarithmic likelihood is abbreviated as “logLik”. Akaike’s information criterion corrected for small sample size (AICc) is used for model selection, such as the Bayesian information criterion (BIC). The delta shows the difference between the AICc values. (PDF) [file pone.0175127.s006.pdf]

S3 Table: Model selection table for testing landscape within groups of different origin (rural and urban). Seven sets of models were run which compared the intercept only model ("Response_null") and a model which include the Origin as explanatory variable (model called as response). The response variables describe the landscape within a buffer around each sample location. Human associated landscape variables (grey) are Sealing (percentage of sealed surface), houses (percentage of houses) and HumDens (Human density per km^2); Forest associated landscape variables (green) are Deciduous and Coniferous (percentage of each forest type); Agricultural associated landscape variables (yellow) are Grassland and Agriculture (percentage of each type).

The degree of freedom is abbreviated as "df". The logarithmic likelihood is abbreviated as "logLik". Akaike's information criterion corrected for small sample size (AICc) is used for model selection, such as the Bayesian information criterion (BIC). The delta shows the difference between the AICc values.

**Model**	**Intercept**	**Origin**	**df**	**logLik**	**AICc**	**delta**	**BIC**
Sealing	2.08	+	5	-884.98	1780.21	0.00	1797.50
Sealing_null	3.49		4	-888.36	1784.88	4.67	1798.75
Houses	2.58	+	5	-1046.89	2104.04	0.00	2121.33
Houses_null	9.07		4	-1054.89	2117.95	13.91	2131.82
HumDens	6.83	+	5	-820.74	1651.74	0.00	**1669.03**
HumDens_null	7.33		4	-821.97	1652.11	0.38	**1665.98**
Deciduous	67.93	+	5	-1040.68	2091.61	0.00	2385.28
Deciduous_null	67.17		4	-1042.55	2093.27	1.65	2399.05
Coniferous	67.93	+	5	-1040.68	2091.61	0.00	2034.53
Coniferous_null	67.17		4	-1042.55	2093.27	1.65	2039.06
Grassland	13.88	+	5	-1004.61	2019.48	0.00	2036.77
Grassland_null	8.98		4	-1011.36	2030.89	11.42	2044.76
Agriculture	13.55	+	5	-879.98	1770.21	0.00	1787.51
Agriculture_null	7.85		4	-899.63	1807.43	37.22	1821.31
