# Supplementary material for: Wild inside: Urban wild boar select natural, not anthropogenic food resources
Source: PLoS One. 2017 Apr 12;12(4):e0175127. doi: 10.1371/journal.pone.0175127 (PMC5389637; doi:10.1371/journal.pone.0175127)
Supplement: S4 Table — Human associated landscape variables (grey) are Sealing (percentage of sealed surface), houses (percentage of houses) and HumDens (Human density per km2); Forest associated landscape variables (green) are Deciduous and Coniferous (percentage of each forest type); Agricultural associated landscape variables (yellow) are Grassland and Agriculture (percentage of each type). Significance (bolt numbers) between urban and rural categories is given, when lower and upper 95% confidence interval (CI) have the same sign (both + or both -). (PDF) [file pone.0175127.s007.pdf]

S4 Table: Tukey posthoc test for models testing landscape within groups of different origin (rural and urban, Table S2, Figure 3): The response variables describe the landscape within a buffer around each sample location. Human associated landscape variables (grey) are Sealing (percentage of sealed surface), houses (percentage of houses) and HumDens (Human density per km²); Forest associated landscape variables (green) are Deciduous and Coniferous (percentage of each forest type); Agricultural associated landscape variables (yellow) are Grassland and Agriculture (percentage of each type). Significance (bolt numbers) between urban and rural categories is given, when lower and upper 95% confidence interval (CI) have the same sign (both + or both -).

**Response**	**Quantile**	**Estimate**	**lower 95% CI**	**upper 95% CI**
Sealing	1.96	2.64	**0.39**	**4.89**
Houses	1.96	15.32	**6.86**	**23.78**
HumDens	1.96	0.82	-0.94	2.59
Desiduous	1.96	33.56	**22.85**	**44.26**
Coniferous	1.96	-10.81	**-19.68**	**-1.92**
Grassland	1.96	-11.02	**-18.07**	**-3.98**
Agriculture	1.96	-16.23	**-21.37**	**-11.07**
