# Supplementary material for: Wild inside: Urban wild boar select natural, not anthropogenic food resources
Source: PLoS One. 2017 Apr 12;12(4):e0175127. doi: 10.1371/journal.pone.0175127 (PMC5389637; doi:10.1371/journal.pone.0175127)
Supplement: S5 Table — Seven sets of models were run which compared the intercept only model (“Response_null”) and a model which include the Origin as explanatory variable (model called as response). The response variables are energy amount of each stomach content (measured in KJ/g dry matter); modulus of fineness (MOF, calculated after particle size determination); the acid insoluble ash (AIA given in percent), such as amount of protein, starch, fat and fibre. The degree of freedom is abbreviated as “df”, the logarithmic likelihood is abbreviated as “logLik”. Akaike’s information criterion corrected for small sample size (AICc) is used for model selection, such as the Bayesian information criterion (BIC). The delta shows the difference between the AICc values. (PDF) [file pone.0175127.s008.pdf]

S5 Table: Model selection table for linear mixed models, testing the impact of origin and stomach category on the nutrient composition of wild boar stomachs. Seven sets of models were run which compared the intercept only model ("Response_null") and a model which include the Origin as explanatory variable (model called as response). The response variables are energy amount of each stomach content (measured in KJ/g dry matter); modulus of fineness (MOF, calculated after particle size determination); the acid insoluble ash (AIA given in percent), such as amount of protein, starch, fat and fibre.

The degree of freedom is abbreviated as "df", the logarithmic likelihood is abbreviated as "logLik". Akaike's information criterion corrected for small sample size (AICc) is used for model selection, such as the Bayesian information criterion (BIC). The delta shows the difference between the AICc values.

**Model**	**Intercept**	**Origin**	**Stomach category**	**df**	**logLik**	**AICc**	**delta**	**BIC**
Energy	20.42	+	+	5	-580.76	1180.27	0.00	1211.09
Energy_null	19.29			4	-589.31	1186.79	6.52	1200.66
MOF_null	2.97			4	-193.60	395.37	0.00	409.23
MOF	2.81	+	+	5	-192.03	402.81	7.45	433.63
AIA	4.92	+	+	5	-844.42	1707.59	0.00	1738.41
AIA_null	8.18			4	-854.86	1717.89	10.30	1731.75
Protein	16.24	+	+	5	-734.88	1488.52	0.00	1519.34
Protein_null	17.48			4	-753.97	1516.11	27.59	1529.49
Starch	26.03	+	+	5	-961.52	1941.81	0.00	1972.63
Starch_null	24.10			4	-1000.55	2009.26	67.46	2023.14
Fat	12.67	+	+	5	-844.89	1708.53	0.00	1739.35
Fat_null	8.81			4	-853.03	1714.23	5.70	1728.10
Fibre	8.10	+	+	5	-731.42	1481.59	0.00	1512.41
Fibre_null	9.31			4	-751.61	1511.39	29.80	1525.26
