# Supplementary material for: Wild inside: Urban wild boar select natural, not anthropogenic food resources
Source: PLoS One. 2017 Apr 12;12(4):e0175127. doi: 10.1371/journal.pone.0175127 (PMC5389637; doi:10.1371/journal.pone.0175127)
Supplement: S6 Table — Significance (bolt numbers) between urban and rural categories is given, when lower and upper 95% confidence interval (CI) have the same sign (both + or both -). (PDF) [file pone.0175127.s009.pdf]

S6 Table: Tukey posthoc test for models testing of landscape within groups of different origin (rural and urban, Table S4, Figure 4): The response variables are energy amount of each stomach content (measured in KJ/g dry matter); modulus of fineness (MOF, calculated after particle size determination); the acid insoluble ash (AIA given in percent), such as amount of protein, starch, fat and fibre. Significance (bolt numbers) between urban and rural categories is given, when lower and upper 95% confidence interval (CI) have the same sign (both + or both -).

Response	Variable	Compared categories	Quantile	Estimate	lower 95% CI	upper 95% CI
Energy	Origin	rural-urban	1.96	0.90	**0.17**	**1.63**
	Stomach category	Acorn fibre- Acorn	2.71	-1.53	**-2.84**	**-2.1**
	Stomach category	Fibre- Acorn	2.71	-2.13	**-3.81**	**-0.45**
	Stomach category	Maize- Acorn	2.71	-1.87	-3.74	0.01
	Stomach category	Mix- Acorn	2.71	-1.66	**-3.116**	**-0.17**
	Stomach category	Fibre-Acorn fibre	2.71	-0.60	-2.01	0.80
	Stomach category	Maize- Acorn fibre	2.71	-0.34	-1.99	1.30
	Stomach category	Mix- Acorn fibre	2.71	-0.13	-1.32	1.05
	Stomach category	Maize- fibre	2.71	0.26	-1.62	2.13
	Stomach category	Mix- Fibre	2.71	0.47	-1.06	2.00
	Stomach category	Mix- Maize	2.71	0.21	-1.55	1.97
MOF	*no data, since best model was null model*					
AIA	Origin	rural-urban	1.96	-1.42	-4.02	1.18
	Stomach category	Acorn fibre- Acorn	2.71	3.51	-0.34	7.35
	Stomach category	Fibre- Acorn	2.71	7.76	**2.98**	**12.54**
	Stomach category	Maize- Acorn	2.71	0.78	-4.62	6.20
	Stomach category	Mix- Acorn	2.71	3.77	-0.56	8.09
	Stomach category	Fibre-Acorn fibre	2.71	4.25	**0.26**	**8.25**
	Stomach category	Maize- Acorn fibre	2.71	-2.72	-7.46	2.01
	Stomach category	Mix- Acorn fibre	2.71	0.26	-3.18	3.70
	Stomach category	Maize- fibre	2.71	-6.97	**-12.46**	**-1.49**
	Stomach category	Mix- Fibre	2.71	-3.99	-8.44	0.45
	Stomach category	Mix- Maize	2.71	2.98	-2.13	8.09
Protein	Origin	rural-urban	1.96	2.37	-0.45	5.19
	Stomach category	Acorn fibre- Acorn	2.71	-0.94	-3.46	1.57
	Stomach category	Fibre- Acorn	2.71	4.07	**0.97**	**7.16**
	Stomach category	Maize- Acorn	2.71	-2.45	-6.01	1.10
	Stomach category	Mix- Acorn	2.71	1.36	-1.39	4.10
	Stomach category	Fibre-Acorn fibre	2.71	5.01	**2.31**	**7.70**
	Stomach category	Maize- Acorn fibre	2.71	-1.51	-4.72	1.69
	Stomach category	Mix- Acorn fibre	2.71	2.30	**0.07**	**4.54**
	Stomach category	Maize- fibre	2.71	-6.52	**-9.97**	**-3.08**
	Stomach category	Mix- Fibre	2.71	-2.71	-5.52	0.11
	Stomach category	Mix- Maize	2.71	3.81	**0.51**	**7.12**

Starch	Origin	rural-urban	1.96	-2.41	-5.95	1.13
	Stomach category	Acorn fibre- Acorn	2.71	1.44	-4.75	7.63
	Stomach category	Fibre- Acorn	2.71	-13.15	**-20.81**	**-5.49**
	Stomach category	Maize- Acorn	2.71	16.47	**7.80**	**25.14**
	Stomach category	Mix- Acorn	2.71	-3.33	-10.27	3.60
	Stomach category	Fibre-Acorn fibre	2.71	-14.59	**-20.98**	**-8.19**
	Stomach category	Maize- Acorn fibre	2.71	15.03	**7.44**	**22.61**
	Stomach category	Mix- Acorn fibre	2.71	-4.77	-10.29	0.75
	Stomach category	Maize- fibre	2.71	29.62	**20.80**	**38.44**
	Stomach category	Mix- Fibre	2.71	9.81	**2.67**	**16.96**
	Stomach category	Mix- Maize	2.71	-19.80	**-28.00**	**-11.60**
Fat	Origin	rural-urban	1.96	1.56	-0.86	3.98
	Stomach category	Acorn fibre- Acorn	2.71	-3.94	**-7.78**	**-0.10**
	Stomach category	Fibre- Acorn	2.71	-5.95	**-10.83**	**-1.07**
	Stomach category	Maize- Acorn	2.71	-6.75	**-12.22**	**-1.28**
	Stomach category	Mix- Acorn	2.71	-5.20	**-9.57**	**-0.83**
	Stomach category	Fibre-Acorn fibre	2.71	-2.01	-6.11	2.10
	Stomach category	Maize- Acorn fibre	2.71	-2.80	-7.61	1.99
	Stomach category	Mix- Acorn fibre	2.71	-1.26	-4.73	2.21
	Stomach category	Maize- fibre	2.71	-0.80	-6.27	4.67
	Stomach category	Mix- Fibre	2.71	0.74	-3.73	5.21
	Stomach category	Mix- Maize	2.71	1.55	-3.60	6.69
Fibre	Origin	rural-urban	1.96	-0.60	-3.43	2.24
	Stomach category	Acorn fibre- Acorn	2.71	1.05	-1.43	3.53
	Stomach category	Fibre- Acorn	2.71	5.05	**2.00**	**8.11**
	Stomach category	Maize- Acorn	2.71	-2.77	-6.27	0.73
	Stomach category	Mix- Acorn	2.71	1.46	-1.25	4.16
	Stomach category	Fibre-Acorn fibre	2.71	4.01	**1.35**	**6.67**
	Stomach category	Maize- Acorn fibre	2.71	-3.82	**-6.98**	**-0.65**
	Stomach category	Mix- Acorn fibre	2.71	0.41	-1.79	2.61
	Stomach category	Maize- fibre	2.71	-7.82	**-11.22**	**-4.43**
	Stomach category	Mix- Fibre	2.71	-3.10	**-6.37**	**-0.82**
	Stomach category	Mix- Maize	2.71	4.23	**0.97**	**7.49**
