# Supplementary material for: Wild inside: Urban wild boar select natural, not anthropogenic food resources
Source: PLoS One. 2017 Apr 12;12(4):e0175127. doi: 10.1371/journal.pone.0175127 (PMC5389637; doi:10.1371/journal.pone.0175127)
Supplement: S7 Table — Only models with an AICc value below 2 were used to calculate the variable importance. Only variables above 0.4 are used for the final model and visualized in Fig 4. (PDF) [file pone.0175127.s010.pdf]

S7 Table: Relative variable importance for linear mixed models, testing nutrient values and food quality in stomachs of wild boar from Berlin and Brandenburg between 2012 and 2015. Only models with an AICc value below 2 were used to calculate the variable importance. Only variables above 0.4 are used for the final model and visualized in Figure 4.

Explanatory Variable <br>**Response**	***Sealing***	***Houses***	***Human Density***	***Deciduous***	***Coniferous***	***Agriculture***	***Grassland***
**Energy-rural**	**1,00**	0.39	0.39				
**Energy-urban**			0.12	0.18	0.15	0.12	0.13
**MOF**	0.18	0.18	**0.44**		**0.40**		0.16
**AIA**						**0.76**	0.28
**Protein**	0.28	0.28	0.28	0.28	**1.00**	0.28	0.28
**Starch**	0.29	0.29	0.29	0.29	0.29	**1.00**	**1.00**
**Fat**			0.24			0.21	**0.76**
**Fibre**					0.18	0.16	**0.57**
