# Supplementary material for: Wild inside: Urban wild boar select natural, not anthropogenic food resources
Source: PLoS One. 2017 Apr 12;12(4):e0175127. doi: 10.1371/journal.pone.0175127 (PMC5389637; doi:10.1371/journal.pone.0175127)
Supplement: S8 Table — For different response variables, the energy amount of each stomach content was measured in KJ/g dry matter. Only the analysis of energy was split into urban and rural origin because Fig 3 showed a significant difference between urban and rural wild boar only for energy. The modulus of fineness (MOF) was calculated after particle size determination; the acid insoluble ash (AIA) is given in percent, such as amount of protein, starch, fat and fibre. The explanatory variables describe the landscape within a buffer around each sample location and were grouped regarding their expected influence: Sealing (percentage of sealed surface), houses (percentage of houses) and HumDens (Human density per km2) are human associated landscape variables (grey). The Models, which include only these variables, are called “Hum1”-“Hum4”. Deciduous (percentage of deciduous forest) and Coniferous (percentage of coniferous forest) are forest associated landscape variables (green); the models which include only these variables are called “For1”-“For3”. Grassland (percentage of grassland) and Agriculture (percentage of agriculture) are agricultural associated landscape variables (shaded in yellow); the model which include only these variables are called “Agr1”-“Agr3”. The full model includes all variables; the intercept only model is called “null”. The degree of freedom is abbreviated as “df”. The logarithmic likelihood is abbreviated as “logLik”. Akaike’s information criterion corrected for small sample size (AICc) is used for model selection, such as the Bayesian information criterion (BIC). The delta shows the difference between the AICc values. (PDF) [file pone.0175127.s011.pdf]

S8 Table: Full model selection table for linear mixed models, testing nutrient values and food quality in stomachs of wild boar from Berlin and Brandenburg between 2012 and 2015. For different response variables, the energy amount of each stomach content was measured in KJ/g dry matter. Only the analysis of energy was split into urban and rural origin because figure 3 showed a significant difference between urban and rural wild boar only for energy. The modulus of fineness (MOF) was calculated after particle size determination; the acid insoluble ash (AIA) is given in percent, such as amount of protein, starch, fat and fibre.

The explanatory variables describe the landscape within a buffer around each sample location and were grouped regarding their expected influence: Sealing (percentage of sealed surface), houses (percentage of houses) and HumDens (Human density per km²) are human associated landscape variables (grey). The Models, which include only these variables, are called “Hum1”-“Hum4”. Deciduous (percentage of deciduous forest) and Coniferous (percentage of coniferous forest) are forest associated landscape variables (green); the models which include only these variables are called “For1”-“For3”. Grassland (percentage of grassland) and Agriculture (percentage of agriculture) are agricultural associated landscape variables (shaded in yellow); the model which include only these variables are called “Agr1”-“Agr3”. The full model includes all variables; the intercept only model is called “null”.

The degree of freedom is abbreviated as “df”. The logarithmic likelihood is abbreviated as “logLik”. Akaike’s information criterion corrected for small sample size (AICc) is used for model selection, such as the Bayesian information criterion (BIC). The delta shows the difference between the AICc values.

**Response**	**Model**	**Intercept**	**sealing**	**houses**	**human density**	**deciduous**	**coniferous**	**grassland**	**agriculture**	**df**	**logLik**	**AICc**	**delta**
Energy rural	Hum2	19.14	0.67							5	-234.87	480.41	0.00
	Hum1	19.21	0.75	-0.41	-0.38					7	-233.01	481.29	0.88
	Hum3	19.25		-0.50						5	-236.26	483.18	2.77
	null	18.98								4	-237.58	483.60	3.19
	Agr2	19.29						-0.47		5	-236.58	483.83	3.42
	Agr3	19.10							0.46	5	-236.81	484.28	3.87
	Agr1	19.25						-0.44	0.42	6	-235.83	484.60	4.19
	For2	19.06				0.32				5	-236.97	484.60	4.19
	For3	18.95					-0.17			5	-237.43	485.53	5.11
	Hum4	18.98			-0.14					5	-237.47	485.61	5.20

	For1	19.04				0.30	-0.07			6	-236.94	486.83	6.42
	Full	19.16	0.68	-0.35	-0.32	-0.01	-0.26	-0.26	0.17	11	-232.37	489.88	9.47
Energy urban	null	19.40								4	-341.78	691.83	0.00
	For2	19.27				-0.21				5	-341.21	692.83	1.00
	For3	19.38			0.18					5	-341.35	693.11	1.28
	Agr2	19.47					-0.15			5	-341.48	693.38	1.55
	Agr3	19.47		-0.13						5	-341.58	693.57	1.74
	Hum4	19.39	0.10							5	-341.63	693.68	1.85
	Hum2	19.40							0.05	5	-341.74	693.89	2.06
	Hum3	19.39						0.03		5	-341.76	693.94	2.11
	For1	19.26			0.15	-0.19				6	-340.89	694.37	2.54
	Agr1	19.54		-0.14			-0.16			6	-341.25	695.09	3.26
	Hum1	19.38	0.09					0.03	0.02	7	-341.61	698.00	6.17
	Full	19.40	0.10	-0.23	0.15	-0.77	-0.52	-0.46	0.02	11	-337.94	699.77	7.94
MOF	For3	2.98					-0.08			5	-191.46	393.16	0.00
	Hum4	2.96			0.06					5	-191.89	394.02	0.86
	Hum1	2.96	-0.04	0.05	0.07					7	-190.14	394.76	1.59
	Agr2	2.96						0.05		5	-192.35	394.95	1.79
	For1	2.98				-0.01	-0.08			6	-191.41	395.18	2.02
	Hum3	2.96		0.05						5	-192.47	395.19	2.03
	null	2.97								4	-193.60	395.37	2.21
	Agr1	2.97						0.05	-0.04	6	-191.83	396.01	2.85
	Agr3	2.97							-0.04	5	-193.08	396.41	3.25
	Full	2.98	-0.04	0.07	0.07	0.07	-0.03	0.07	0.00	11	-186.80	396.72	3.56
	Hum2	2.97	-0.02							5	-193.47	397.19	4.03
	For2	2.97				0.01				5	-193.53	397.30	4.14
AIA	Agr3	8.48							-1.10	5	-853.16	1716.57	0.00
	Agr1	8.42						0.53	-1.11	6	-852.61	1717.58	1.01
	null	8.18								4	-854.86	1717.89	1.32
	Agr2	8.14						0.50		5	-854.39	1719.02	2.45
	For3	8.01					0.71			5	-854.55	1719.35	2.78
	Hum4	8.19			0.29					5	-854.69	1719.63	3.06
	Hum2	8.17	-0.28							5	-854.69	1719.63	3.07
	Hum3	8.18		0.21						5	-854.77	1719.79	3.22
	For2	8.13				-0.15				5	-854.83	1719.90	3.33
	For1	7.98				-0.13	0.68			6	-854.53	1721.40	4.84
	Hum1	8.17	-0.40	0.21	0.41					7	-854.29	1723.04	6.48
	Full	8.35	-0.35	0.25	0.35	0.30	0.42	0.71	-0.92	11	-852.12	1727.35	10.79
Protein	For3	18.01					-1.35			5	-749.85	1509.95	0.00

	Full	18.06	-0.31	-1.00	0.14	-1.54	-2.27	-0.78	-1.12	11	-744.35	1511.82	1.87
	For1	17.98				-0.11	-1.37			6	-749.80	1511.96	2.01
	Agr3	17.61							-0.64	5	-752.42	1515.10	5.15
	null	17.48								4	-753.97	1516.11	6.16
	Agr1	17.61						0.18	-0.64	6	-752.28	1516.90	6.95
	Hum2	17.46	-0.20							5	-753.77	1517.79	7.85
	Agr2	17.48						0.18		5	-753.81	1517.88	7.93
	Hum3	17.48		-0.05						5	-753.96	1518.17	8.22
	Hum4	17.49			0.03					5	-753.97	1518.18	8.24
	For2	17.49				0.03				5	-753.97	1518.18	8.24
	Hum1	17.46	-0.23	-0.05	0.10					7	-753.72	1521.91	11.96
Starch	Agr1	24.10						-1.80	2.14	6	-995.49	2003.33	0.00
	Full	24.10	0.64	1.96	-1.01	3.89	3.57	0.05	2.90	11	-991.01	2005.15	1.82
	Agr3	24.10							2.14	5	-997.61	2005.47	2.14
	For1	24.10				1.55	2.69			6	-996.65	2005.65	2.32
	For3	24.10					2.02			5	-997.93	2006.11	2.78
	Agr2	24.11						-1.80		5	-998.48	2007.20	3.87
	null	24.10								4	-1000.55	2009.26	5.93
	Hum4	24.10			-0.78					5	-1000.16	2010.57	7.24
	Hum3	24.10		-0.61						5	-1000.32	2010.88	7.55
	For2	24.10				0.39				5	-1000.45	2011.15	7.82
	Hum2	24.10	-0.02							5	-1000.55	2011.35	8.02
	Hum1	24.10	0.24	-0.63	-0.87					7	-999.87	2014.22	10.89
Fat	Agr2	9.00						-1.00		5	-850.98	1712.22	0.00
	Hum4	8.82			0.75					5	-851.83	1713.90	1.69
	Agr1	8.89						-1.00	0.23	6	-850.89	1714.13	1.92
	null	8.81								4	-853.03	1714.23	2.02
	Hum2	8.83	0.68							5	-852.08	1714.41	2.19
	Hum3	8.85		-0.30						5	-852.86	1715.96	3.75
	Agr3	8.68							0.27	5	-852.90	1716.06	3.84
	For2	8.83				0.04				5	-853.03	1716.31	4.10
	For3	8.82					-0.04			5	-853.03	1716.31	4.10
	Full	8.87	0.44	-1.13	0.65	-1.94	-1.60	-1.90	-0.37	11	-846.87	1716.86	4.64
	Hum1	8.87	0.49	-0.27	0.60					7	-851.21	1716.88	4.67
	For1	8.83				0.03	-0.03			6	-853.03	1718.41	6.19
Fibre	Agr2	9.30						0.57		5	-750.08	1510.41	0.00
	null	9.31								4	-751.61	1511.39	0.98
	For3	9.55					-0.55			5	-750.93	1512.11	1.69
	Agr1	9.33						0.57	-0.15	6	-749.99	1512.33	1.92

For2	9.19				-0.35				5	-751.16	1512.56	2.15
For1	9.44				-0.41	-0.63			6	-750.29	1512.93	2.52
Agr3	9.34							-0.16	5	-751.52	1513.28	2.87
Hum3	9.31		0.10						5	-751.57	1513.39	2.97
Hum2	9.32	0.08							5	-751.58	1513.41	2.99
Hum4	9.31			0.06					5	-751.60	1513.45	3.03
Hum1	9.33	0.08	0.10	0.03					7	-751.53	1517.52	7.11
Full	9.45	0.01	-0.28	0.09	-0.53	-0.62	0.26	-0.31	11	-749.35	1521.83	11.42
